# A Landscape of Murine Long Non-Coding RNAs Reveals the Leading Transcriptome Alterations in Adipose Tissue during Aging

**DOI:** 10.1016/j.celrep.2020.107694

**Published:** 2020-05-26

**Authors:** Qiuzhong Zhou, Qianfen Wan, Yuxi Jiang, Jin Liu, Li Qiang, Lei Sun

**Affiliations:** 1 Cardiovascular and Metabolic Disorders Program, Duke-NUS Medical School, 8 College Road, Singapore 169857, Singapore; 2 Naomi Berrie Diabetes Center, Department of Pathology and Cell Biology, College of Physicians and Surgeons, Columbia University, New York, NY 10032, USA; 3 Zhejiang Provincial Key Laboratory of Medical Genetics, Key Laboratory of Laboratory Medicine, Ministry of Education, Wenzhou, Zhejiang 325035, China; 4 School of Laboratory Medicine and Life Sciences, Wenzhou Medical University, Wenzhou, Zhejiang 325035, China; 5 Centre for Quantitative Medicine, Health Services & Systems Research, Duke-NUS Medical School, 8 College Road, Singapore 169857, Singapore; 6 Institute of Molecular and Cell Biology, 61 Biopolis Drive, Proteos, Singapore 138673, Singapore; 7 These authors contributed equally; 8 Lead Contact

## Abstract

Aging is an inevitable process that involves profound physiological changes. Long non-coding RNAs (lncRNAs) are emerging as important regulators in various biological processes but are not systemically studied in aging. To provide an organism-wide lncRNA landscape during aging, we conduct comprehensive RNA sequencing (RNA-seq) analyses across the mouse lifespan. Of the 1,675 aging-regulated lncRNAs (AR-lncRNAs) identified, the majority are connected to inflammation-related biological pathways. AR-lncRNAs exhibit high tissue specificity; conversely, those with higher tissue specificity are preferentially regulated during aging. White adipose tissue (WAT) displays the highest number of AR-lncRNAs and develops the most dynamic crosstalk between AR-lncRNA and AR-mRNA during aging. An adipose-enriched AR-lncRNA, lnc-adipoAR1, is negatively correlated with aging, and knocking it down inhibits adipogenesis, phenocopying the compromised adipogenic capacity of aged fat. Our works together reveal AR-lncRNAs as essential components in aging and suggest that although each tissue ages in a distinct manner, WAT is a leading contributor to aging-related health decline.

## In Brief

Zhou et al. generated a comprehensive RNA-seq dataset of 11 tissues throughout the mouse lifespan, identified thousands of aging-regulated lncRNAs, and revealed leading transcriptome alterations in adipose tissue during aging. One of the AR-lncRNAs, lnc-AdipoAR1, regulates adipogenesis. This study serves as a valuable resource for further studies on lncRNAs during aging.

## Graphical Abstract

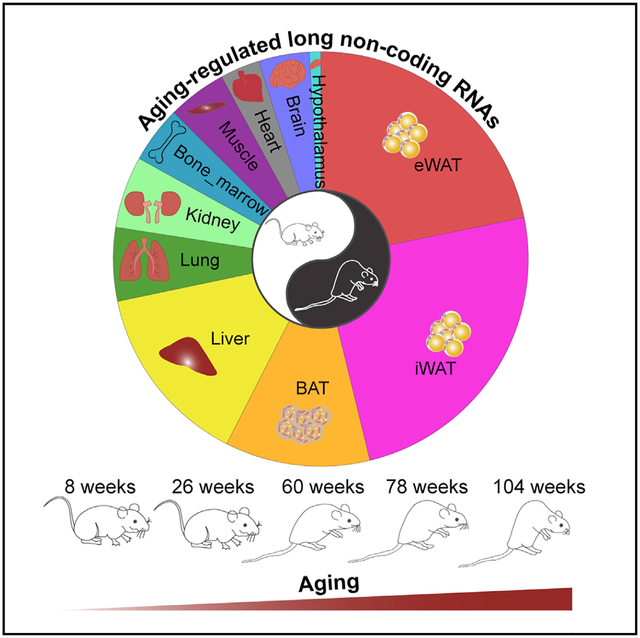

## INTRODUCTION

Aging is an inevitable physiological process in which molecular and cellular damage accumulate, leading to health decline and increased vulnerability to disease and death (Aunan et al., 2016; Melzer et al., 2020). Aging is the predominant risk factor for prevalent diseases including obesity, diabetes, cardiovascular diseases, cancer, and neurodegeneration (Franceschi et al., 2018; Niccoli and Partridge, 2012). The proportion of the global aged population (above 60 years) is predicted to increase from 10.0% in 2000 to 21.8% in 2050, which poses a severe challenge for both public health and research (Lutz et al., 2008). Understanding the mechanisms underlying the aging process is a prerequisite for developing lifestyle or pharmaceutical interventions to enhance human healthspan and quality of life.

Transcriptome change is a significant molecular signature of aging and serves as an essential factor in the aging-associated functional decline across organs (Benayoun et al., 2019; Braun et al., 2016; Schaum et al., 2019; Stoeger et al., 2019; Yu et al., 2014; Zahn et al., 2007). Several earlier studies have profiled and analyzed age-correlated gene expression in different organs in rodents (Barns et al., 2014; Benayoun et al., 2019; Braun et al., 2016; de Magalhães et al., 2009; Ori et al., 2015; Schaum et al., 2019; Stoeger et al., 2019; Yu et al., 2014; Zahn et al., 2007; Zhuang et al., 2019) and humans (Ahadi et al., 2020; Glass et al., 2013; Wang et al., 2018; Yang et al., 2015; Zhuang et al., 2019). The majority of the genes altered during aging are associated with inflammatory responses (Benayoun et al., 2019; Schaum et al., 2019; Stegeman and Weake, 2017), highlighting chronic inflammation as a hallmark of aging. Interestingly, most aging-associated genes manifest tissue-specific regulation, and only a small fraction of genes are commonly regulated in multiple organs (Benayoun et al., 2019; Schaum et al., 2019; Zahn et al., 2007), demonstrating that aging is an organ-specific process (Ori et al., 2015).

While earlier studies have mainly focused on protein-coding genes, the function of non-coding RNAs during aging remains poorly understood. About two-thirds of the mammalian genome is pervasively transcribed, but only 2%–3% of the genome encodes proteins (Fatica and Bozzoni, 2014; Wilusz et al., 2009). The majority of the genome is transcribed into non-coding transcripts, the main category of which is long non-coding RNAs (lncRNAs), an emerging class of players in various biological processes (BPs) including aging (Cao et al., 2019; Grammatikakis et al., 2014; Kim et al., 2016; Kour and Rath, 2016; Xing et al., 2017). For example, the lncRNA *Bmncr* regulates the osteogenic niche alteration and fate switch of bone marrow mesenchymal stem cells during skeletal aging (Li et al., 2018). Additionally, the lncRNA *NEAT1* is involved in neurodegeneration, and inhibiting it in the hippocampus improves the memory of elderly mice through the repression of neuronal histone methylation (Butler et al., 2019). With the emerging role of lncRNAs in aging, a barrier that hinders further functional and mechanistic studies is the lack of an organism-wide landscape of aging-regulated lncRNAs (AR-lncRNAs).

In this study, we performed comprehensive analyses of the regulation of lncRNAs during the functional decline of multiple murine tissues over time, leading to the identification of a class of AR-lncRNAs. We found that white adipose tissue (WAT) had the highest number of AR-lncRNAs and displayed the most dynamic AR-lncRNA~AR-mRNA crosstalk evolved during aging among all examined organs, strongly suggesting that adipose tissue is a leading contributor to the organismal decline. Our study thereby serves as a valuable resource and provides a framework for further functional and mechanistic studies on lncRNAs during aging.

## RESULTS AND DISCUSSION

### Global Transcriptome Characterization of Multiple Tissues during Aging

**Figure 1.**
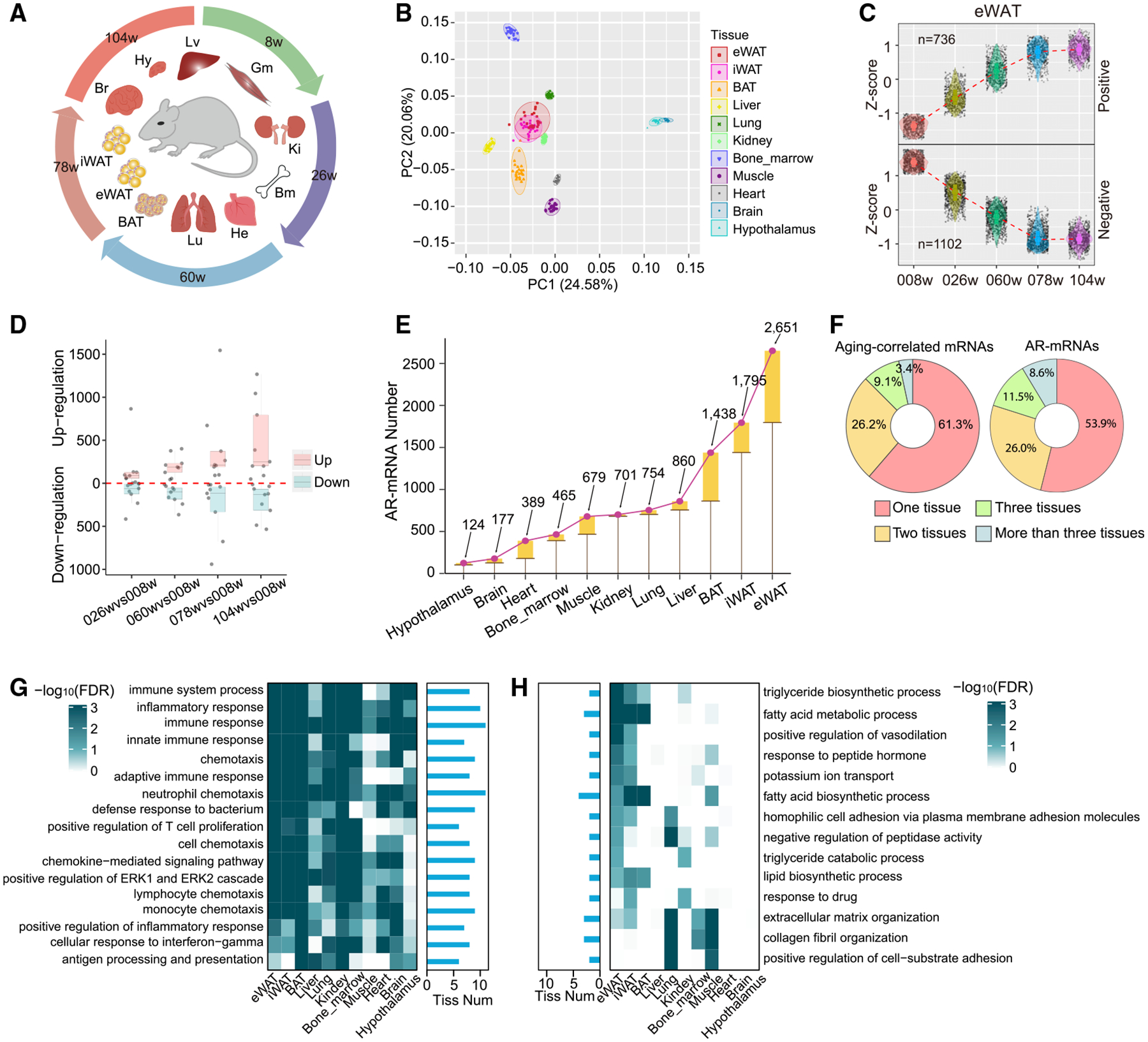
Global Transcriptome Characterization of Multiple Tissues during Aging (A) Eleven tissues (brain, Br; hypothalamus, Hy; lung, Lu; bone marrow, Bm; gastric muscle, Gm; liver, Lv; kidney, Ki; heart, He; inguinal white adipose tissue, iWAT, iW; epididymal white adipose tissue, eWAT, eW; and brown adipose tissue, BAT, Ba) were collected at five different stages across the mouse lifespan. (B) Principal component analysis (PCA) for 275 samples based on all gene expression. (C) The expression pattern of aging-correlated mRNAs in eWAT during aging. Gene expression is normalized by *Z*-score across the five age stages. (D) Number of differentially expressed mRNAs by comparing the elder groups (26, 60, 78, and 104 weeks) to the younger group (8 weeks). The numbers of upregulated or downregulated mRNAs across all tested tissues were split into quartiles. The box covers a range from the first quartile (Q1) to the third quartile (Q3). The second quartile (Q2) was indicated by a vertical line in the box. (E) Number of aging-regulated mRNAs (AR-mRNAs) across all tested organs. The height of the boxes indicates the increased number of AR-mRNAs for the corresponding tissue in comparison to the left-neighbor tissue. (F) Percent of the aging-correlated mRNAs (left) and AR-mRNAs (right) that are identified in one, two, three, and more than three organs. (G and H) Biological processes (BPs) that are commonly enriched in at least six different organs in the upregulated AR-mRNAs (G) and commonly enriched in at least two different organs in the downregulated AR-mRNAs (H). Histogram indicates the number of tissues with functional enrichment for the corresponding BP.

To systemically profile the transcriptome changes during aging in an organism-wide manner, we conducted RNA sequencing (RNA-seq) at five different stages (8, 26, 60, 78, and 104 weeks) across the mouse lifespan and in 11 different organs including the brain, hypothalamus, lung, bone marrow, gastric muscle, liver, kidney, heart, inguinal WAT (iWAT), epididymal WAT (eWAT), and brown adipose tissue (BAT) (Figure 1A). We mapped~12 billion pair-end reads from 275 samples against the mouse genome (GRCm38) to quantify the expression of both mRNAs and lncRNAs. A gene with FPKM (fragments per kilobase per million) > 0.5 in at least 20% of samples is considered detectable in our dataset. Using such a criterion, we detected 12,315~16,060 qualified genes across the examined tissues (Table S1). Principal component analysis (PCA) (Figure 1B) shows that these transcriptomes are grouped largely according to their organ identity instead of their aging stage, indicating that organ identity is still the predominant factor determining gene expression during aging.

To examine how the aging process may affect gene expression across different tissues, we defined aging-induced genes with two different approaches. In the first approach, we analyzed the correlative relationship between gene expression and aging over time, which led to the identification of hundreds of positively and negatively correlated genes in each organ (Figures 1C and S1A). In another more stringent approach, we determined the differentially expressed genes (DEGs) by comparing the two aged groups (78 and 104 weeks) to the younger group (8 weeks), with a cutoff of q value < 0.1 and log_2_ fold-change > 0.75, leading to the identification of ~5,550 AR mRNAs (AR-mRNAs) in the whole organism (Figure 1D; Table S2), with the highest numbers in eWAT (2,651) and iWAT (1,795) (Figure 1E; Table S2). In both approaches, these AR genes were largely identified in an organ-specific manner (Figures S1B and S1C). Only a small portion of them were identified in more than three tissues (Figure 1F; Table S2), further supporting the organ-specific nature of aging.

The most pronounced pathways enriched by these AR genes are associated with immune response, and this feature is recapitulated in multiple tissues (Figure 1G). Of note, the pathways associated with the genes downregulated during aging are more organ specific and are often related to the organ’s biological function (Figure 1H). For example, the processes of triglyceride biosynthesis, fatty acid metabolism, and fatty acid biosynthesis are particularly enriched in the aging-repressed genes in three adipose tissue depots (eWAT, iWAT, and BAT) but not in other organs (Figure 1H), indicating a functional decline of lipid metabolism in adipose tissue during aging.

### Identification of AR-lncRNAs

**Figure 2.**
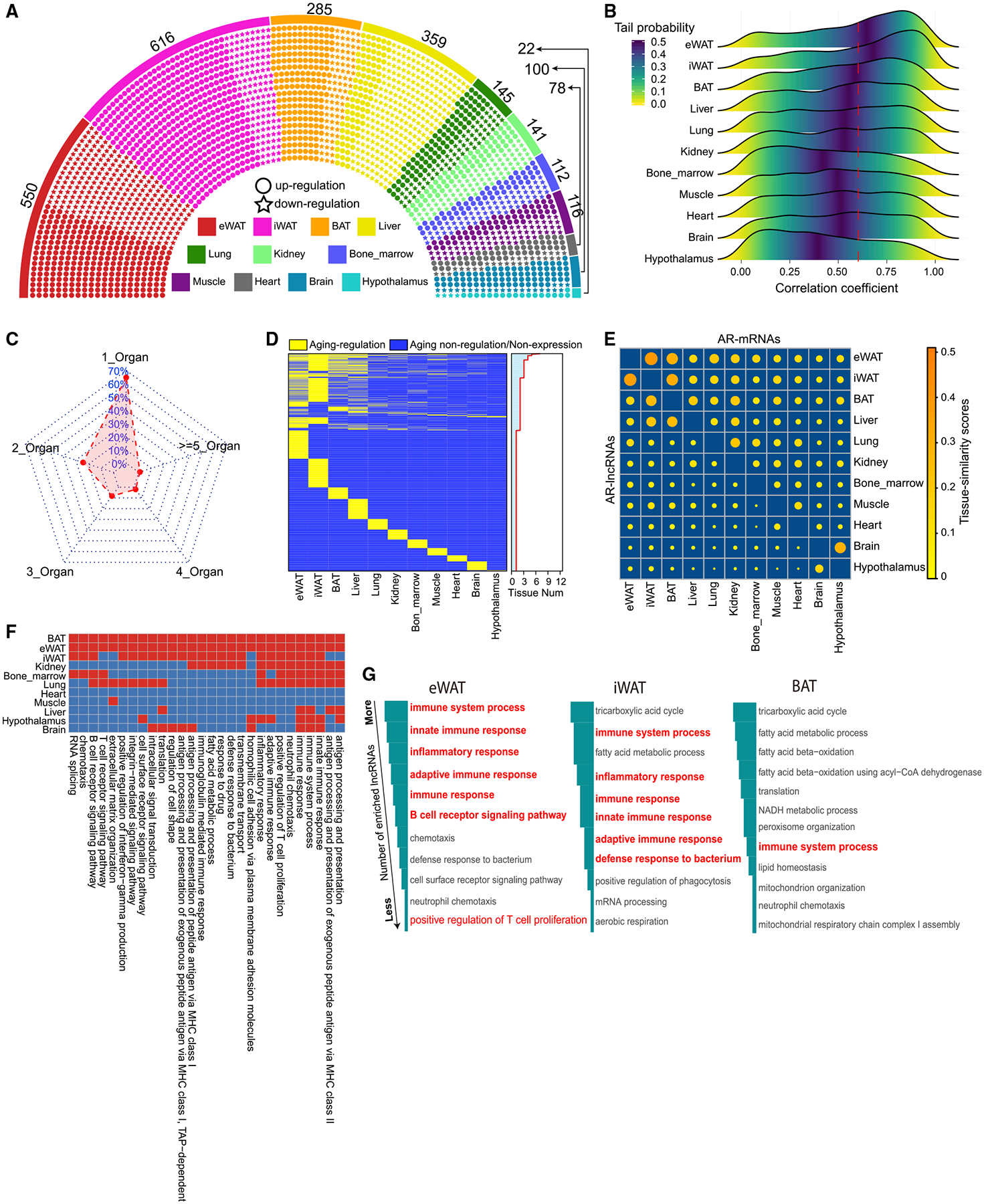
Identification and Functional Annotation of Aging-Regulated lncRNAs (A) The distribution of aging-regulated lncRNAs (AR-lncRNAs) in all examined organs. Each dot represents an AR-lncRNA. (B) The distribution of correlation coefficient between lncRNA expression and age in each tissue. (C) Percentage of AR-lncRNAs uniquely discovered in a single organ and commonly discovered in multiple organs. (D) The presence of AR-lncRNAs across all examined tissues. Distribution in right panel represents the number of tissues in which a given AR-lncRNA can be identified. (E) The tissue-similarity scores of AR-lncRNAs and AR-mRNAs between different tissues in a pairwise manner. Top right triangle: the tissue-similarity scores of AR-mRNAs. Bottom left triangle: the tissue-similarity scores of AR-lncRNAs. Tissue-similarity scores represent the tissue similarity between two organs based on the overlapped extent of their gene sets (detail in STAR Methods). (F) BPs that are associated with lncRNAs through lncRNA~mRNA co-expression analysis in more than three tissues. (G) The top BPs ranked by the number of AR-lncRNAs that are associated with each BP in adipose tissues.

The organ-specific nature of aging inspired us to further examine the dynamic landscapes of lncRNAs during aging, because lncRNAs are expressed more cell-type specifically than mRNAs and may significantly contribute to the organ-specific nature of aging. We compared the lncRNA expression from the aged groups (78 and 104 weeks) with that from the younger group (8 weeks) and identified 1,675 AR-lncRNAs (Figure 2A; Table S2). As expected, the AR-lncRNAs, compared with aging-non-regulated lncRNAs (ANR-lncRNAs), showed a stronger correlation with aging course (Figure S2A), and were more dynamically expressed during aging in all 11 examined organs (Figures S2B and S2C). A few previously reported AR-lncRNAs such as *H19* (Hofmann et al., 2019), *Malat1* (Zhu et al., 2019), and *Neat1* (White et al., 2015) were identified in our AR-lncRNA list, attesting to the validity of our data analysis (Table S2). Notably, the two WAT depots (eWAT and iWAT) had the highest number of AR-lncRNAs among all examined organs (Figure 2A; Table S2). eWAT and iWAT expressed 550 and 616 AR-lncRNAs, respectively, while the heart, brain, and hypothalamus had no more than 100 AR-lncRNAs (Figure 2A). Consistent with the AR-lncRNA enrichment in WAT, the global lncRNAs detected in eWAT and iWAT had higher correlation coefficients with aging compared to those in other tissues (Figure 2B). The large numbers of AR-mRNAs (Figure 1E) and AR-lncRNAs (Figure 2A) in eWAT and iWAT suggest that WAT likely undergoes more dramatic functional alterations than other examined organs and thereby more significantly contributes to organismal decline during aging.

We further investigated the organ-specific nature of aging by examining the number of AR-lncRNAs shared by multiple organs. Approximately 70% of AR-lncRNAs passed our aging-regulation criteria only in a single organ (Figures 2C and 2D), while only ~30% of AR-lncRNAs were commonly regulated in more than one organ (Figure 2C). The majority of the common AR-lncRNAs were found in eWAT, iWAT, BAT, or liver (Figures 2D and S2D), likely due to their functional conjunction in metabolism. In comparison with AR-mRNAs, the AR-lncRNAs exhibited lower tissue-similarity scores (Figures 2E and S2E). To exclude the effect of abundance difference between lncRNAs and mRNAs on the tissue-similarity analysis, we examined the tissue-similarity scores of a subset of lncRNAs and a subset of mRNAs with matched expression abundance and still observed lower tissue-similarity scores in AR-lncRNAs (Figures S2F and S2G). Thus, the lower tissue-similarity scores of AR-lncRNAs are not merely due to the biotype expression difference, and AR-lncRNAs indeed better reflect the organ-specific nature of aging.

To explore the potential functional role of these AR-lncRNAs, we conducted co-expression analysis between AR-lncRNAs and all mRNAs in each tissue (Table S3). For each AR-lncRNA, we analyzed the BP enrichment of its correlated mRNAs as an indicator for the AR-lncRNA’s function. At the organismal level, the predominately enriched BPs are immune-response related, such as the immune system processes, innate immune responses, and inflammatory responses (Figure S2H). These immune-related BPs are also predominant among those that overlap in multiple tissues (Figure 2F). At the tissue level, AR-lncRNAs in WAT are more closely associated with immune-related BPs than other examined tissues. For instance, eWAT had 174 AR-lncRNAs linked to immune-related BPs (Figure S2I; Table S3), which was the highest among all examined tissues. Ranking by the number of lncRNAs in each BP, the top BPs in eWAT and iWAT were primarily related to immune response (Figure 2G). Taken together, our lncRNA~mRNA co-expression analysis demonstrated that AR-lncRNAs are functionally connected to immune response at the organismal level, and such connections are particularly strong in WAT.

### The Tissue Specificity of Aging-Dependent Regulation of AR-lncRNAs

**Figure 3.**
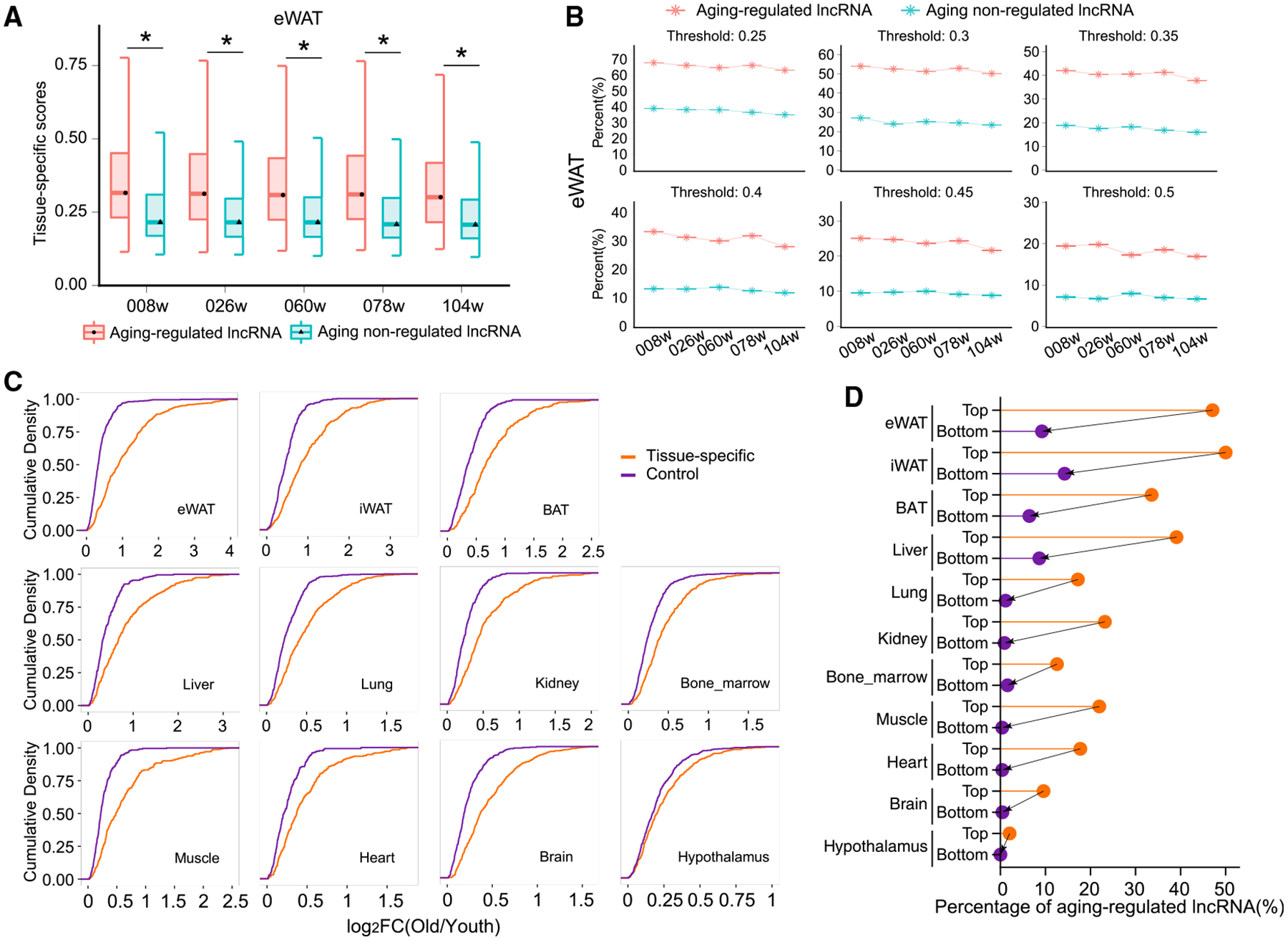
The Aging-Regulatory Feature of lncRNAs Interacts with Their Tissue-Specific Nature (A) The tissue-specific scores of AR-lncRNAs and aging-non-regulated lncRNAs (ANR-lncRNAs) across the mouse lifespan. The result from eWAT is shown as representative data. *p < 0.05, Mann-Whitney test. (B) Percentage of tissue-specific lncRNAs in AR-lncRNAs and ANR-lncRNAs under varying thresholds of tissue-specific score. The result from eWAT is shown as representative data. (C) Cumulative density of log_2_(old/young) between tissue-specific and control lncRNAs in all examined tissues. Log_2_FC(old/young) is the max log_2_(fold change, FC) between 78-week and 8-week and between 104-week and 8-week samples. The tissue-specific and control lncRNAs are top 20% and bottom 20% lncRNAs ranked by lncRNAs’ tissue-specific scores at a certain age point. The results from 8-week-old samples are shown as representative data. (D) Percentage of AR-lncRNAs in the tissue-specific and control lncRNAs across all tested tissues. The result from 8-week samples is shown as representative data.

To characterize the AR-lncRNAs in comparison with other lncRNAs, we assessed their molecular and genetic features including gene length, isoform number, exon number, and expression abundance in each tissue, but we did not observe any consistent differences across all examined organs (Figures S3A–S3D). In contrast, AR-lncRNAs had significantly higher tissue-specificity scores than other lncRNAs across all five age points (Figures 3A and S3E). To examine this feature more quantitatively, we classified lncRNAs as tissue-specific lncRNAs using six different maximal fraction thresholds. Regardless of the threshold employed, the AR-lncRNAs had a higher percentage of tissue-specific lncRNAs than other lncRNAs across all age stages (Figures 3B and S3F). Thus, AR-lncRNAs are expressed in a more tissue-specific manner.

To investigate whether tissue-specific lncRNAs are responsive to aging, at each age point, we ranked all lncRNAs according to their tissue-specific scores, defined the top 20% as the tissue-specific lncRNAs and the bottom 20% as the universally expressed controls, and compared their dynamic changes during aging. The tissue-specific lncRNAs exhibited more dynamic expression changes during aging across all examined organs (Figures 3C and S4A–S4D). A higher rate of the tissue-specific lncRNAs is AR-lncRNAs across all tested tissues (Figures 3D and S4E). Therefore, the tissue-specific lncRNAs show stronger aging-associated regulation.

### WAT Develops Interwoven AR-lncRNA~AR-mRNA Crosstalk during Aging

**Figure 4.**
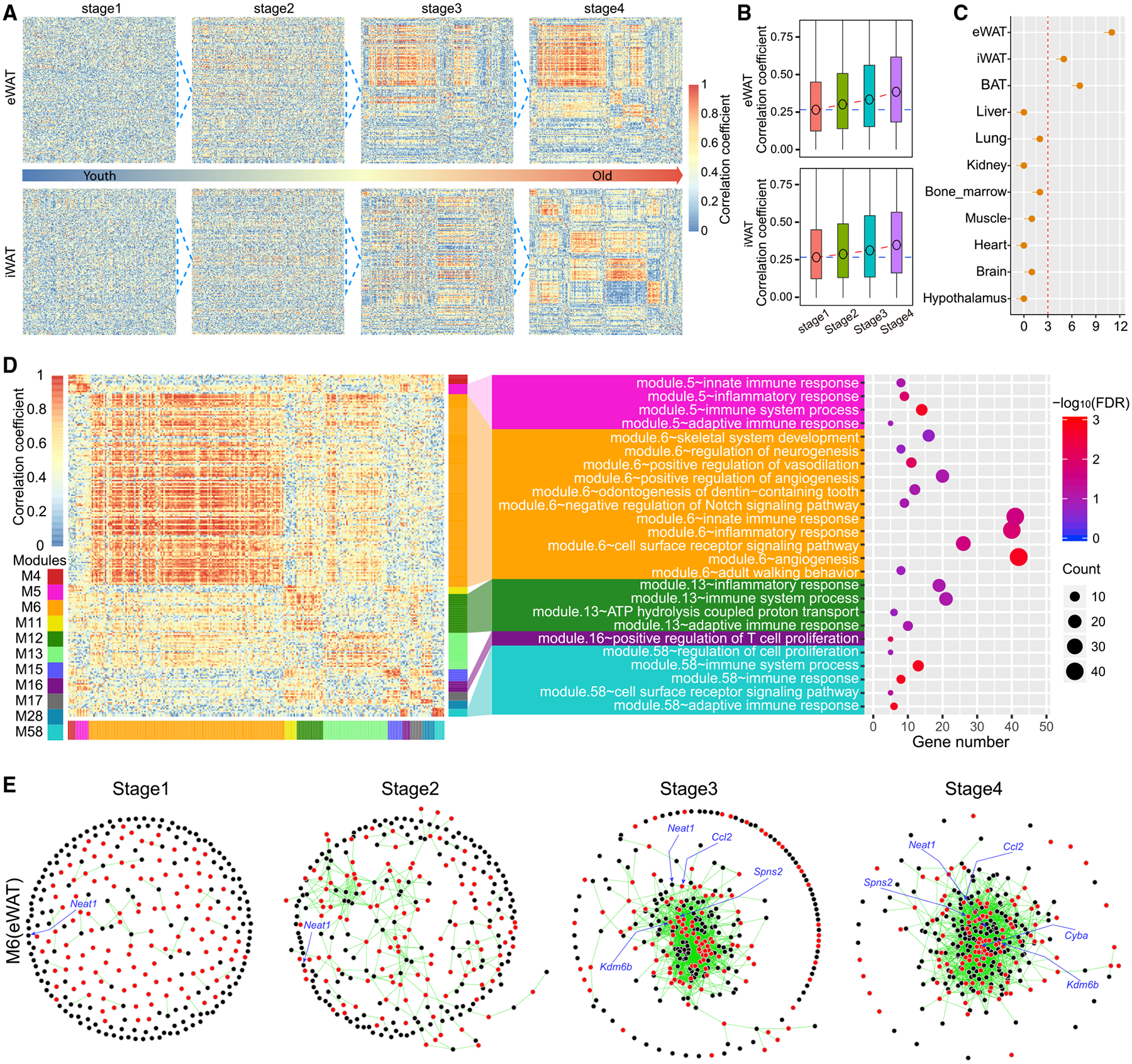
WAT Develops Dynamic AR-lncRNA~AR-mRNA Crosstalk during Aging (A) The heatmap of networks between AR-lncRNAs and AR-mRNAs during aging in eWAT and iWAT. Each row represents an AR-lncRNA, while each column represents an AR-mRNA. The color code represents the correlation coefficient between each AR-lncRNA and AR-mRNA comparison across different age stages. The 1, 2, 3, and 4 stages include samples from 8- and 26-, 26- and 60-, 60- and 78-, and 78- and 104-week samples, respectively. (B) The global correlation coefficients between AR-lncRNAs and AR-mRNAs at different aging stages in eWAT (upper) and iWAT (lower). (C) The number of mRNA~lncRNA modules with more than 30 genes (AR-lncRNAs and AR-mRNAs) in each tissue. (D) The heatmap for all consensus modules with more than 30 genes in eWAT between the stage 1 and stage 4 (left) and functional enrichment of genes in each module (middle). The number of genes involved in each biological pathway (right). (E) The dynamic change of AR-lncRNA~AR-mRNA interactions for genes involved in inflammation pathways in the largest module (M6) of eWAT.

A correlative expression between different genes suggests that they may share common upstream regulators, directly or indirectly regulate each other, or participate in similar biological functions. Thus, correlated genes tend to form networks governing functionally related pathways. The growth of these networks often reflects the enhanced functional and regulatory interplays between different components. We constructed the AR-lncRNA~AR-mRNA networks and analyzed their dynamic changes during aging in each individual tissue (Figure S5A). These networks exhibited great heterogeneity with respect to size and growth across all examined tissues (Figures 4A and S5B). The networks in adipose tissue grew more drastically during aging (Figure 4A) than those in other organs (Figure S5B). The overall AR-lncRNA~AR-mRNA correlations increased gradually and persistently in eWAT and iWAT (Figure 4B) but not significantly in other tissues (Figure S5C). The eWAT, iWAT, and BAT contained 11, 5, and 7 significant AR-lncRNA~AR-mRNA modules with > 30 genes, respectively, but none of the other examined organs had more than 3 modules (Figure 4C). It is notable that network changes in the brain, a widely appreciated driver of aging, mostly occur at the very late stage of aging (Figure S5C), while those network changes in eWAT and iWAT occur earlier during aging (Figure 4A), supporting a driving role of adipose tissue during aging.

To assess what BPs the AR networks in adipose tissue may regulate, we conducted Gene Ontology (GO) analysis on the AR-lncRNA~AR-mRNA modules that progressively grew during aging. Most genes in eWAT modules were associated with inflammatory response pathways (Figure 4D). The AR-lncRNA~AR-mRNA connections involved in these pathways grew in both number and strength (Figure 4E). *Neat1*, a previously reported lncRNA that regulates neuronal histone methylation during aging (Butler et al., 2019), was embodied in the center of the largest AR-lncRNA~AR-mRNA module in eWAT (Figure 4E). In iWAT, we observed a similar growth of AR-lncRNA~AR-mRNA networks during aging, which was connected to inflammatory response and lipid metabolism (Figures S5D and S5E). Taken together, our analysis demonstrates that AR-lncRNAs and AR-mRNAs in adipose tissue become closely intertwined during aging to form networks that regulate inflammation-related processes.

### Lnc-AdipoAR1 Knockdown Inhibits Adipogenesis

**Figure 5.**
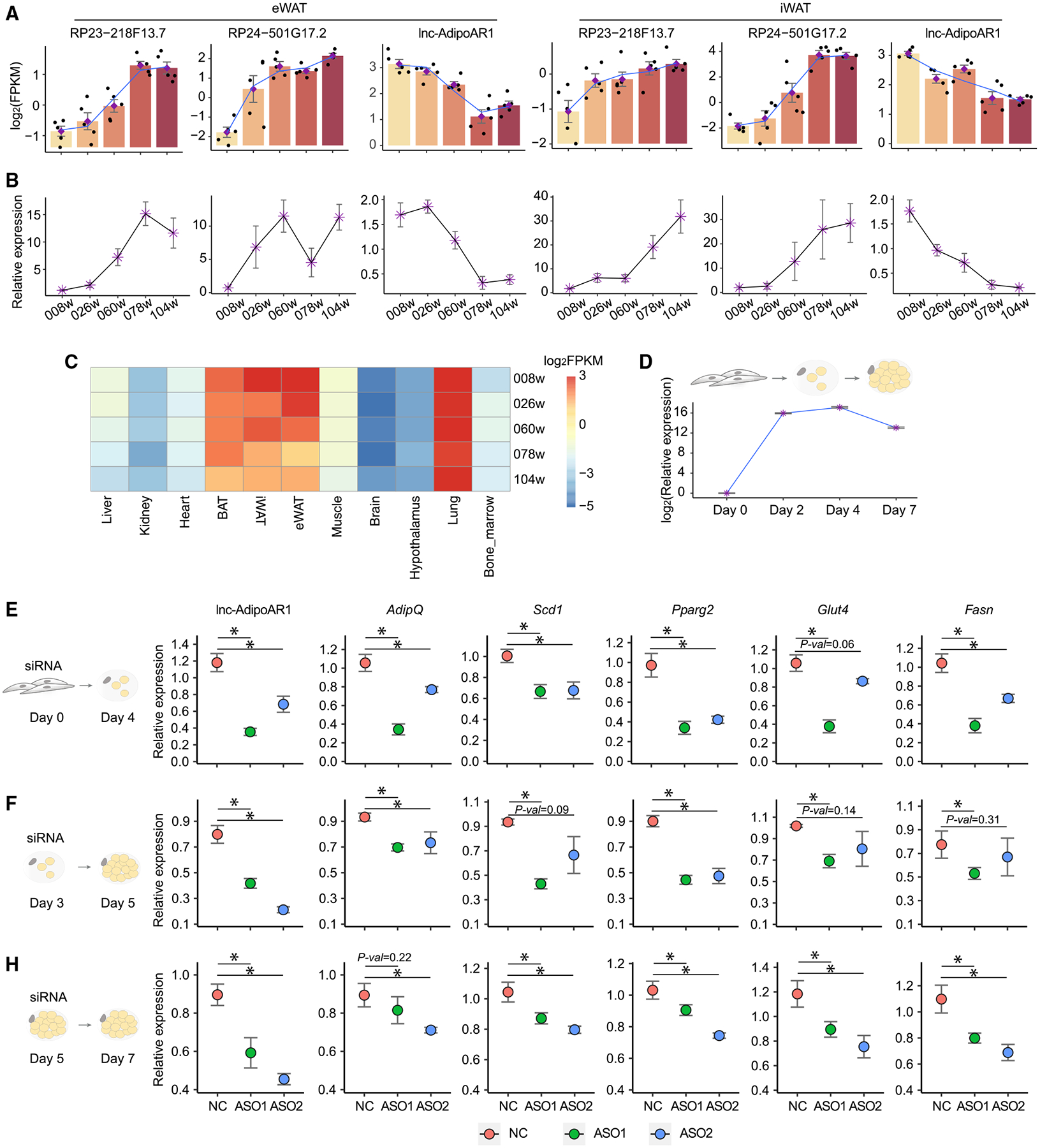
Lnc-AdipoAR1 Knockdown Inhibits the Expression of Adipogenesis Markers (A) RNA-seq expression of RP23–218F13.7, RP24–501G17.2, and lnc-AdipoAR1 (AC116511.4) in eWAT and iWAT during aging. (B) Real-time PCR validations of RP23–218F13.7, RP24–501G17.2, and lnc-AdipoAR1 in eWAT and iWAT during aging. The expression of each gene was normalized to house-keeping gene Cyclophilin A (*CPA*). n = 5; error bars are mean ± SEM. (C) The expression of lnc-AdipoAR1 across different tissues during aging. (D) Real-time PCR analysis of lnc-AdipoAR1 during adipogenesis of 3T3-L1 adipocytes. The expression of lnc-AdipoAR1 was normalized to *RPL-23*. n = 4; error bars are mean ± SEM. (E–G) lnc-AdipoAR1 was knocked down with ASOs in iWAT-derived adipocyte culture at day 0 (E), day 3 (F), or day 5 (G) during adipogenesis. RNA was harvested at indicated timing for real-time PCR to examine the expression of indicated adipocyte markers. Gene expression was normalized to *RPL-23*. n = 4 (E and F) and n = 3 (G); error bars are mean ± SEM, *p < 0.05, Student’s t test.

Since the above analyses have suggested a driving role of WAT in aging, we further focused on the AR-lncRNAs in adipose tissues for expression validation and functional analysis. We identified 207 AR-lncRNAs common in eWAT and iWAT (Table S4) and selected three of them based on their abundance and p values (RP23–218F13.7, RP24–501G17.2, and AC116511.4) for real-time PCR validation. All of them can be successfully validated and show similar expression patterns during aging between RNA-seq and real-time PCR, attesting to our dataset analysis (Figures 5A and 5B; Table S5). AC116511.4 is particularly interesting because it is enriched in adipose tissue, upregulated during adipogenesis, but downregulated during aging (Figures 5A–5D; Table S5). It is referred to as adipose AR-lncRNA 1 (lnc-AdipoAR1) below. To explore the function of lnc-AdipoAR1 in adipocytes, we used antisense oligos (ASOs) to knock it down in primary adipocyte culture at day 0, day 3, and day 5 during adipogenesis. Regardless of the time points, the knockdown of lnc-AdipoAR1 consistently reduced the expression of adipocyte markers such as *AdipQ*, *Scd1*, and *Pparg2* (Figures 5E–5G; Table S5), indicating a critical role of this lncRNA in adipogenesis. Because the declined adipogenesis is a hallmark of aged adipose tissue (Caso et al., 2013; Karagiannides et al., 2001; Palmer and Kirkland, 2016), we postulate that the reduced expression of lnc-AdipoAR1 is likely to contribute to the impaired adipogenic capacity in aging.

In summary, we have comprehensively profiled the lncRNA dynamic changes during aging by analyzing 275 samples from 11 tissues at five age points across the mouse lifespan, leading to the identification of 1,675 AR-lncRNAs. Through the identification of lncRNAs, our study reinforces the tissue-specific and inflammatory nature of aging. Our study reveals a remarkable number of AR-lncRNAs (Figure 2A; Table S2) as well as AR-mRNAs (Figure 1E; Table S2) in WAT and a significant growth of the AR-lncRNA~AR-mRNA networks during aging. In agreement with earlier reports that adipose tissue is one of the few organs where tissue-restricted interventions can impact healthspan and lifespan (Schaum et al., 2019), our study has demonstrated that AR-lncRNAs and AR-mRNAs may underlie the driving role of WAT changes in aging.

## STAR⋆METHODS

### RESOURCE AVAILABILITY

#### Lead Contact

Further information and requests for resources and reagents should be directed to lead contact, Dr. Lei Sun (sun.lei@duke-nus.edu.sg).

#### Materials Availability

This study did not generate new unique reagents.

#### Data and Code Availability

The accession number for the RNA-seq raw data reported in this paper is NGDC: PRJCA002140.

### EXPERIMENTAL MODEL AND SUBJECT DETAILS

C57BL/6J male mice at the age of 8, 26, 60, 78, and 104 weeks were purchased from Jackson Laboratory and housed in the ventilated animal barrier at Columbia University with temperature set to 23 ± 1°C, 12 h day/light cycle, and free access to food and water. The animal experiments are approved by the Columbia University Animal Care and Utilization Committee (New York, NY, USA).

### METHOD DETAILS

#### Samples collection

C57BL/6J animals were obtained from Jackson Laboratory. 5 mice at 5 different age points (8wk = human 20yr, 26wk = human 34yr, 60wk = human 60yr, 78wk = human 65yr, and 104wk = human 70yr) from across mouse lifespan were sacrificed to harvest 11 types of tissues including brain, hypothalamus, lung, bone marrow, gastric muscle, liver, kidney, heart, inguinal white adipose tissue (iWAT), epididymal white adipose tissue (eWAT), and brown adipose tissue (BAT) (Dutta and Sengupta, 2016). A total of 275 tissue samples were prepared for RNA sequencing.

#### RNA extraction

Fresh tissues were immediately immersed into TriZol reagent (Thermo Fisher) and processed with homogenization. Following chloroform phase separation, RNA from the upper clear layer were further extracted by using RNA isolation kit from Macherey-Nagel according to the manufacturer’s instructions.

#### RNA sequencing

The strand-specific RNA-seq libraries were prepared and sequenced in Novogene. The quality of libraries was assessed Agilent 2100. RNA-seq libraries were multiplexed and RNA sequencing were performed with the 150 bp pair-end reads on the HiSeq X ten platform.

Total ~12 billion pair-end reads and ~2TB data (fastq.gz files) were generated from the 275 RNA-seq libraries. Median and average of RNA sequencing depth are ~44 and ~45 million pair-end reads, respectively. The 5% and 95% quantiles of sequencing depth are ~ 41 million and ~52 pair-end reads, respectively. The sequencing depth was steady across the 275 RNA-seq samples.

#### Quantification of gene expression

Quality control of RNA-seq data was carried out with fastqc (v.0.11.2) (Andrews, 2010). The pair-end reads from each sample were aligned against the mouse reference genome (Release M17 of GenCode, GRCm38) using STAR (v.2.6.0c) with the parameter-sjdbOverhang 149 (Dobin et al., 2013). The read alignment was guided by the known gene annotation (Release M17 of GenCode) (Dobin et al., 2013). The reference genome sequence was downloaded from the release M17 of GENCODE (https://www.gencodegenes.org) (Frankish et al., 2019). The alignment files were sorted using the samtools (Li et al., 2009). FeatureCounts (v.1.6.3) was employed to compute the read counts of mRNAs and lncRNAs (Liao et al., 2014). The multiply mapping reads were excluded. The read counts were normalized by TMM (Trimmed Mean of M-values) method and converted to FPKM (fragments per kilobase per million) using the R package, edgeR (v.3.20.9) (Robinson et al., 2010). The FPKMs were transformed to log_2_ values for downstream analysis. We filtered out the lowly expressed genes and only remain the gene with FPKM > 0.5 in at least 20% samples which are considered detectable.

#### Identification of aging-correlated genes

We calculated the Pearson correlation coefficient between gene expression (log_2_(FPKM)) and age points (log_2_(weeks)) for each gene in every tissue (Schaum et al., 2019). The gene with a Pearson correlation coefficient > 0.9 and *P-*value < 0.05 was defined as the aging-correlated gene. Expression pattern of aging-correlated genes was plotted using the R graphics package, ggplot2 v.3.2.0 (Wickham, 2016).

#### Tissue-similarity score

We adopted the equation in Enrichment map to assess the connections between different gene sets and to define the tissue-similarity score between differential tissues (Merico et al., 2010). The tissue-similarity score was calculated using the following equation:

Tissue similarity=0.8*U+0.2*I

**U** is the **u**nion of the two gene sets of two differential tissues and **I** is the intersection of these two gene sets.

#### Functional enrichment analysis

We used the DAVID v6.8 (https://david.ncifcrf.gov) to perform the functional enrichment of mRNA (Huang et al., 2009). We downloaded the API program of Perl from DAVID official web site (https://david.ncifcrf.gov/) and used the sub-program, “chartReport_readListsFromFiles.pl” to conduct the functional enrichment analysis. We kept the BPs with background genes % 400 and > = 10 and query genes > = 5, and used a threshold of FDR < 0.1 to define as the significant enrichment of BP (Dhar et al., 2019; Wheeler et al., 2015). Results were visualized by the R package of gplots v.3.0.1.1 (Warnes et al., 2019), ggplot2 v.3.2.0 (Wickham, 2016), and ComplexHeatmap v.2.1.0 (Gu et al., 2016). R packages were run on R version 3.4.5.

#### Identification of aging-regulated genes shared in multi-tissues

To assess the transcriptome changes during aging, we used the criteria of |log_2_FC| > 0.75 and FDR < 0.1 to identify the differentially expressed genes by comparing the older groups (26, 60, 78 and 104 weeks) to the youth group (8 weeks) in each tissue, respectively (Law et al., 2016; Robinson et al., 2010). The tissues from 78 and 104 weeks were regarded as old samples and the genes that were differentially expressed in 078w versus 008w or 104w versus 008w were defined as the aging-regulated genes including the Aging-Regulated mRNAs (AR-mRNAs) and Aging-Regulated lncRNAs (AR-lncRNAs).

#### Functional annotation of aging-regulated lncRNAs

To predict the potential functional role of AR-lncRNAs, we performed the co-expression between all mRNAs and AR-lncRNAs in the samples specified in the text (Necsulea et al., 2014). For each AR-lncRNA, we identified its co-expressed mRNAs with a cutoff at Pearson correlation coefficient > 0.8 and adjust *P value* < 0.05) in each tissue. We then used DAVID to perform the functional annotation of the co-expressed mRNAs for each AR-lncRNA (Huang et al., 2009).

#### Basic features between aging-regulated and aging non-regulated lncRNAs

We investigated the features including the gene length, transcript number, and exon number between AR-lncRNAs and ANR-lncRNAs base on the gene annotation of mouse (Release M17 of GenCode), which was downloaded from the GENCODE (https://www.gencodegenes.org) (Frankish et al., 2019).

#### Age-specific score analysis of lncRNAs

We used the gene expression of 5 age points to assess age-specificity across the examined tissues (Alvarez-Dominguez et al., 2015; Ding et al., 2018). The fractional expression for each lncRNAs (AR-lncRNAs and ANR-lncRNAs) in a given age in a specific tissue was defined as the proportion of its expression against the cumulative expressions of this lncRNA across 5 age points (Alvarez-Dominguez et al., 2015; Ding et al., 2018). In the following equation of fractional expression, *A*_*ij*_ is the average expression of a given gene *i* in the age of *j*. *i* is the gene id and *j* represents the age of the samples.

$$\text{Age fraction}=\frac{Aij}{\sum_{j=1}^{5}A_{ij}}$$

For each lncRNA, the highest age-fraction was used as its age-specific score and the standard deviation calculated by the sd function of R stats package, was used to assess the variation of age-fractions.

#### Tissue-specificity of lncRNAs

We used the gene expression from 11 tissues to evaluate the tissue-specific score across the mouse lifespan (008w, 026w, 060w, 078w, and 104w) (Alvarez-Dominguez et al., 2015; Ding et al., 2018). The fractional expression for each lncRNA (AR-lncRNAs and ANR-lncRNAs) in a given tissue was defined as the proportion of its expression against the cumulative expressions of this lncRNA across all tested tissues (Alvarez-Dominguez et al., 2015; Ding et al., 2018). In the following equation of fractional expression, *T*_*ij*_ is the average expression of a given gene *i* in a given tissue *j*. *i* is the gene id and *j* is the tissue id.

$$\text{Tissue fraction}=\frac{Tij}{\sum_{j=1}^{11}T_{ij}}$$

The fractional expression was calculated at each time point separately. If a given lncRNAs is specifically expressed in a given tissue, this lncRNA will have the maximal fractional expression in this tissue. The highest tissue fraction of a lncRNA was used as its tissue-specific score (Alvarez-Dominguez et al., 2015; Ding et al., 2018). To compare the numbers of tissue-specific lncRNAs in AR-lncRNAs and ANR-lncRNAs, we used different thresholds of tissue-specific scores (0.25, 0.3, 0.35, 0.4, 0.45 and 0.5) to define tissue-specific lncRNAs and compare the percent of tissue-specific lncRNAs between AR-lncRNAs and ANR-lncRNAs (Ding et al., 2018).

#### Aging-regulated changes between tissue-specific and control lncRNAs

We ranked all detectable lncRNAs based on their tissue-specific scores and defined the top 20% as tissue-specific lcnRNAs while the bottom 20% as control lncRNAs. We defined the aging-regulated changes, log_2_FC(old/youth), for each lncRNA as its larger log_2_(Fold Change, FC) between 78-week versus 8-week and 104-week versus 8-week (Shavlakadze et al., 2019). We used the cumulative distributions of log_2_FC(old/young) of tissue-specific and control lncRNAs to compare their aging-regulated changes.

#### Co-expression networks between lncRNAs and mRNAs

We combined the samples from the neighbor age points into 4 stages (stage1: 008w&026w, stage2: 026w&060w, stage3: 060w&078w, and stage4: 078w&104w). We calculated the Z-scores for all detectable genes and then preformed the co-expression analysis based on the Pearson’s correlation using R package Hmisc v.4.2 (http://cran.r-project.org/web/packages/Hmisc) (Harrell, 2019; Schaum et al., 2019). We conducted the co-expression analysis in each tissue and at each stage separately. To study the interplays between AR-lncRNAs and AR-mRNAs during aging, we then constructed the AR-lncRNA~AR-mRNA networks consensus in the young (stage 1) and old stage (stage 4) (Langfelder and Horvath, 2007; Miller et al., 2010). We retained the edges between lncRNAs and mRNAs in these networks with correlation > 0.9 and FDR < 0.05.

We identified all modules more than 30 genes in the consensus networks using igraph v.1.2.4.1 (cluster_walktrap, step = 10) (Csardi and Nepusz, 2006) and further annotated these modules with DAVID 6.8 (https://david.ncifcrf.gov) (Huang et al., 2009). The dynamic network was displayed by ndtv v.0.12.3 (Bender-deMoll, 2016) and pheatmap v.1.0.12 (Kolde, 2019).

#### ASO designment

Anti-sense oligos were purchased from IDT.

**Negative control:** +G*+A*+C*T*A*T*A*C*G*C*G*C*A*+A*+T*+A
**Lnc-AdipoAR1 ASO 1:** +A*+G*+A*A*T*C*C*C*C*A*T*G*T*+T*+G*+G
**Lnc-AdipoAR1 ASO 2:** +A*+G*+G*G*T*A*C*T*G*G*A*C*T*+T*+T*+C
+N = Affinity Plus locked nucleic acid base
* = Phosphorothioate bonds

#### Primer sequences for real-time PCR

**Lnc-AdipoAR F:** TCCCTAAACCACACTCAGCC
**Lnc-AdipoAR R:** GTGAATGTTCGCTAGTTGCCT
**RP23–218F13.7 F:** TCAGTTCTGAGTGCTCCACC
**RP23–218F13.7 R:** CTCACGGAGTGCTGATGACT
**RP24–501G17.2 F:** CCTGTGATCCGTTTCCATTGT
**RP24–501G17.2 R:** AAGGACAGTTTCTGACCTCAA
***CPA* F:** TATCTGCACTGCCAAGACTGAGTG
***CPA* R:** CTTCTTGCTGGTCTTGCCATTCC
***RPL23* F:** TGTGAAGGGAATCAAGGGAC
***RPL23* R:** TGTTTACTATGACCCCTGCG
***AdipoQ* F:** CGATTGTCAGTGGATCTGACG
***AdipoQ* R**: CAACAGTAGCATCCTGAGCCCT
***Scd1* F**: TTCTTGCGATACACTCTGGTGC
***Scd1* R**: CGGGATTGAATGTTCTTGTCGT
***Pparg2* F**: GCATGGTGCCTTCGCTGA
***Pparg2* R**: TGGCATCTCTGTGTCAACCATG
***Glut4* F:** CTGTCGCTGGTTTCTCCAACT
***Glut4* R:** CCCATAGCATCCGCAACATA
***Fasn* F:** GGAGGTGGTGATAGCCGGTAT
***Fasn* R:** TGGGTAATCCATAGAGCCCAG

#### Mouse adipocyte culture and transfection

3T3-L1 preadipocytes were cultured in DMEM with 10% calf serum (CS) until induction of differentiation. Two days post confluence, cells were switched to adipogenic medium (10% FBS, 1 μM dexamethasone, 0.5 mM 3-isobutyl-1-methylxanthine, and 1.67 μM insulin) for two days. Thereafter, cells were maintained in 10% FBS DMEM containing 0.42 μM insulin for maturation with medium change every 2 days.

Preadipocytes’ isolation, culture, and differentiation were conducted as our previous studies (Siang et al., 2020; Sun et al., 2013). Briefly, iWATs from ~3-week-old mice were minced, and digested in 0.2% collagenase (Sigma), which were subsequently filtered by 40 μm cell strainer and centrifuged to collect stromal vascular fraction (SVF) cells in the pellets. SVF cells were cultured for downstream experiments. Primary SVF cells were cultured in DMEM with 10% FBS and 1% penicillin-streptomycin until confluence. Cells were induced to differentiate with DMEM containing 10% FBS, 850 nM insulin (Sigma), 0.5 μM dexamethasone (Sigma), 250 μM 3-isobutyl-1-methylxanthine, phosphodiesterase inhibitor (IBMX, Sigma), and 1 μM rosiglitazone (Cayman Chemical). The day when the induction starts is considered as Day 0. 48 hours after cocktail induction, the induction medium was replaced with DMEM containing 10% FBS and 160 nM insulin for another 48 hours. Then cells were maintained in DMEM with 10% FBS.

Anti-sense oligos (ASOs) (Integrated DNATechnologies) were designed to specifically target lnc-AdipoAR1. Primary white preadipocytes were seeded onto 24-well plates and grow to confluence. Cells were transfected with 100 nM ASOs using 5 μL ml^−1^ Lipofectamine RNAiMAX (ThermoFisher) according to manufacture instruction. Total RNA was harvested 96 h post-transfection for real-time PCR analysis. For the transfection at day 3 and day 5, 200nM ASOs were employed and total RNAs were harvested 48 hours after transfection.

### QUANTIFICATION AND STATISTICAL ANALYSIS

We used the log_2_FPKM value to quantify the expression level of genes and a cutoff of q value < 0.1, log_2_FC > 0.75 to define the differentially expressed genes (Law et al., 2016; Robinson et al., 2010). The details of gene expression analysis can be found in the “METHOD DETAILS.” For the functional enrichment analysis, we used a threshold of FDR < 0.1 to identify the biological process of significant enrichment. We detected the lncRNA~mRNA crosstalk with a criterion of expressed correlation > 0.9 and FDR < 0.05. Differences in tissue-specific scores between AR-lncRNAs and ANR-lncRNAs were analyzed using Mann-Whitney tests. *P*-value < 0.5 have been considered as significant.

## Supplementary Material

Supple Figures

Supple Table S1

Supple Table S2

Supple Table S3

Supple Table S4

Supple Table S5

## Figures and Tables

**Table:** KEY RESOURCES TABLE

REAGENT or RESOURCE	SOURCE	IDENTIFIER
Biological Samples		
C57BL/6J (8 weeks)	The Jackson Laboratory	https://www.jax.org/
C57BL/6J (26 weeks)	The Jackson Laboratory	https://www.jax.org/
C57BL/6J (60 weeks)	The Jackson Laboratory	https://www.jax.org/
C57BL/6J (78 weeks)	The Jackson Laboratory	https://www.jax.org/
C57BL/6J (104 weeks)	The Jackson Laboratory	https://www.jax.org/
Critical Commercial Assays		
NucleoSpin RNA kits	Macherey-Nagel	740406.50
Antisense Oligo	IDT	https://sg.idtdna.com/pages
TRI Reagent	Sigma-Aldrich	T9424
Deposited Data		
RNA-seq raw data are available at NGDC (https://bigd.big.ac.cn/) under the BioProject accession number PRJCA002140	This paper	NGDC: PRJCA002140
Experimental Models: Organisms/Strains		
Mouse: C57BL/6J	The Jackson Laboratory	https://www.jax.org/
Software and Algorithms		
Quality control of RNA-seq: fastqc v.0.11.2	Andrews, 2010	http://www.bioinformatics.babraham.ac.uk/projects/fastqc/
RNA-seq Mapping: STAR v.2.6.0c	Dobin et al., 2013	https://github.com/alexdobin/STAR
Counting reads: featureCounts v.1.6.3	Liao et al., 2014	http://subread.sourceforge.net/
R system: R v3.4.5	N/A	https://cran.r-project.org/
Programming environment of R: RStudio v1.2.5001	N/A	https://rstudio.com/
Differential gene expression analysis: limma v.3.34.9	Law et al., 2016	https://bioconductor.org/packages/release/bioc/html/limma.html
Differential gene expression analysis: edgeR v.3.20.9	Robinson et al., 2010.	https://bioconductor.org/packages/release/bioc/html/edgeR.html
Functional annotation: DAVID v.6.8	Huang et al., 2009	https://david.ncifcrf.gov/
Gene expression interaction: Hmisc v.4.2	Harrell, 2019	https://cran.r-project.org/web/packages/Hmisc/index.html
Network cluster: igraph v.1.2.4.1	Csardi and Nepusz, 2006	https://igraph.org/redirect.html
Network Dynamic Visualizations: ndtv v.0.12.3	Bender-deMoll, 2016	https://github.com/statnet/ndtv
Data visualization: ggplot2 v.3.2.0	Wickham, 2016	https://ggplot2.tidyverse.org/
Data visualization: gplots v.3.0.1.1	Warnes et al., 2019	https://cran.r-project.org/web/packages/gplots/index.html
Data visualization: ComplexHeatmap v.2.1.0	Gu et al., 2016	https://github.com/jokergoo/ComplexHeatmap
Data visualization: pheatmap v.1.0.12	Kolde, 2019	https://github.com/raivokolde/pheatmap

## Highlights

- Gene expression of 11 tissues was profiled throughout the lifespan of mice
- Aging-regulated lncRNAs exhibit more tissue specificity than other lncRNAs
- Adipose tissue is a leading contributor to aging-related transcriptome alterations
- An aging-regulated lncRNA, lnc-AdipoAR1, is essential for adipogenesis
