# Supplementary material for: A Landscape of Murine Long Non-Coding RNAs Reveals the Leading Transcriptome Alterations in Adipose Tissue during Aging
Source: Cell Rep. Author manuscript; Available in PMC 2020 Nov 1. (PMC7603645; doi:10.1016/j.celrep.2020.107694)
Supplement: Supple Figures [file NIHMS1639069-supplement-Supple_Figures.pdf]

Cell Reports, Volume *31*

Supplemental Information

A Landscape of Murine Long Non-Coding RNAs Reveals the Leading Transcriptome Alterations in Adipose Tissue during Aging

Qiuzhong Zhou, Qianfen Wan, Yuxi Jiang, Jin Liu, Li Qiang, and Lei Sun

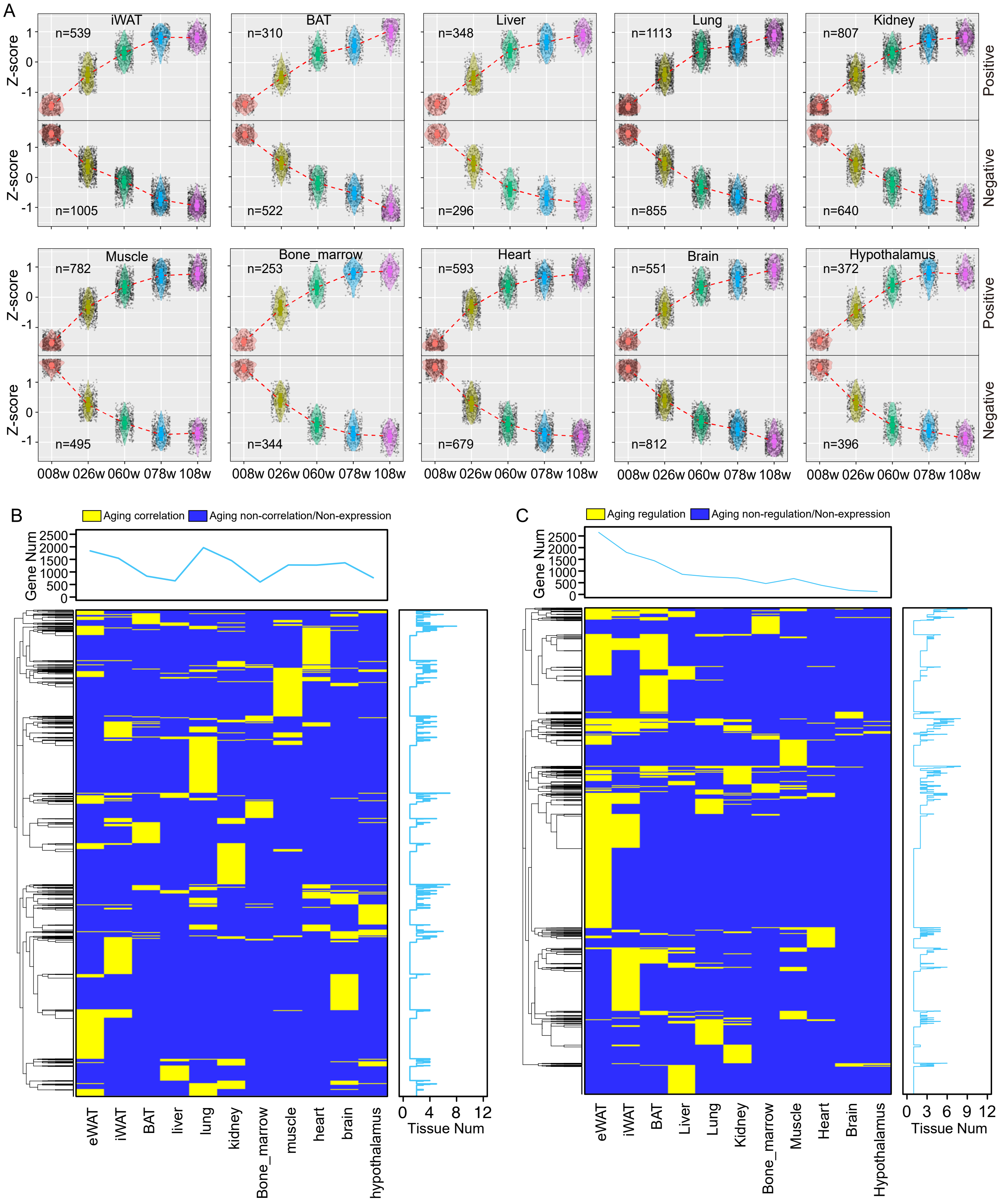

Figure S1. Aging-correlated and aging-regulated mRNAs across all examined organs, (Related to Figure 1)
(A) Expression pattern of the aging-correlated mRNAs in iWAT, BAT, Liver, Lung, Kidney, Muscle, Bone marrow, Heart, Brain, and Hypothalamus. The aging-correlated mRNA was identified by the correlative relationship between mRNA expression and aging time course. Gene expression was normalized by Z-score across the 5 age stages. (B) Aging correlation of aging-correlated mRNAs across all examined organs. The yellow color indicates that a mRNA is identified as aging-correlated in the corresponding tissue while the blue color indicates that the mRNA is not considered as aging-correlated. (C) Aging regulation of aging-regulated lncRNAs across all examined organs. The aging-regulated mRNA was identified via the differential expressed mRNA by comparing the two old groups (78 and 104 weeks) to the young group (8 weeks). The yellow color indicates that a mRNA is identified as aging-regulated in the corresponding tissue while the blue color indicates that the mRNA is not considered as aging-regulated. The distribution of number of aging-correlated mRNAs and aging-regulated mRNAs across all examined organs are indicated in the top panel.

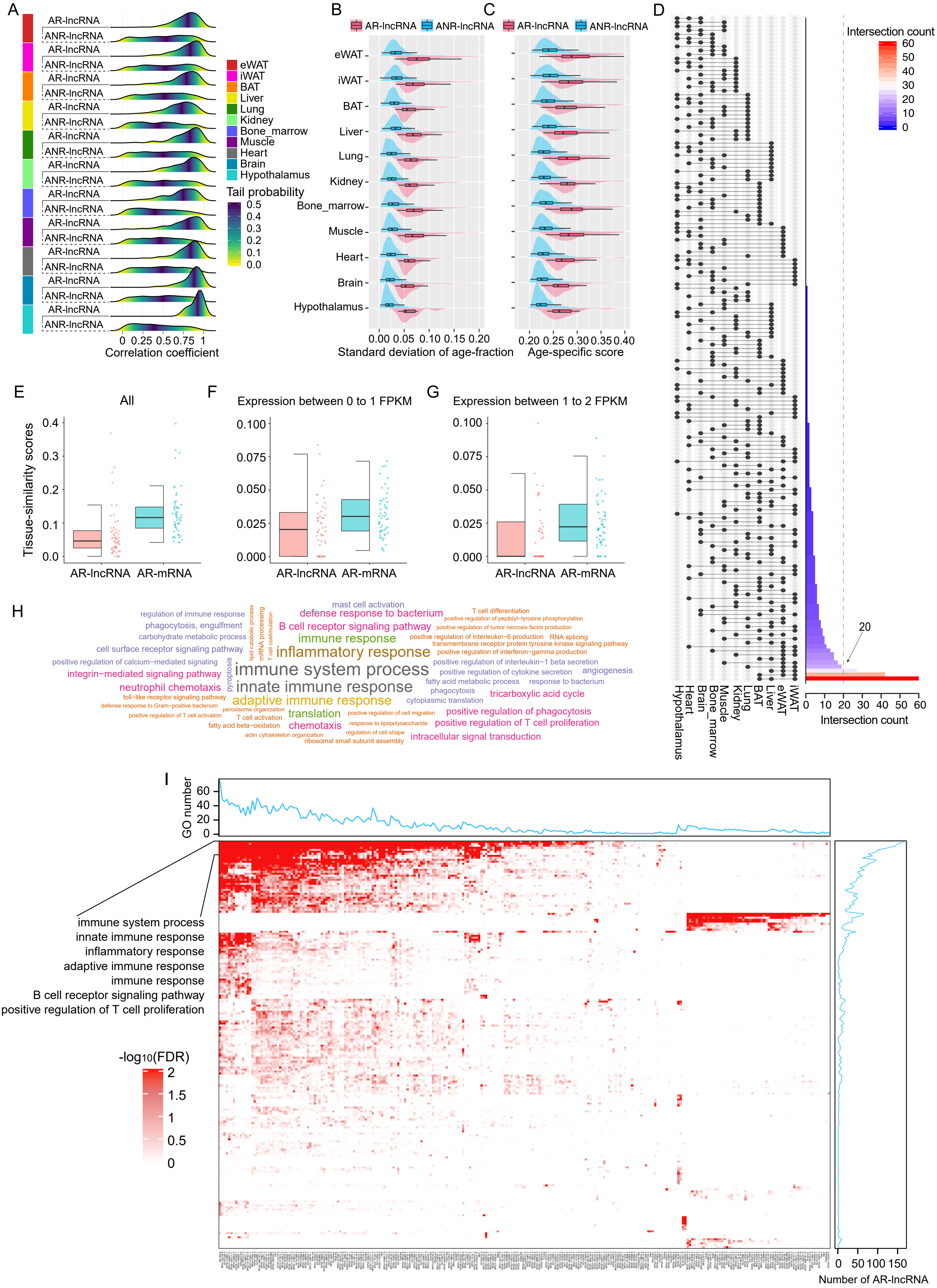

Figure S2. Aging regulation, organ specificity, and functional annotation of AR-lncRNAs, (Related to Figure 2)

(A) The distribution of correlation coefficients between lncRNA expression and age for the AR-lncRNAs and ANR-lncRNAs across all

examined organs. (B) The standard deviation of age-fraction of AR-lncRNAs and ANR-lncRNAs in each organ. (C) Age-specific scores of AR-lncRNAs and ANR-lncRNAs for each organ. (D) Histogram indicates the number of AR-lncRNAs shared in the 3 indicated tissues. (E) The tissue-similarity scores of AR-lncRNAs and AR-mRNAs. (F) The tissue-similarity scores of AR-lncRNAs and AR-mRNAs with the abundance in 0<FPKM<=1 and (G) 1 < FPKM <=2. The tissue-similarity scores were calculated based on the overlapping extent of the AR-lncRNAs between two differential tissues (Detail in STAR Methods). (H) Top BPs ranked by the number of AR-lncRNAs associated with each BP. The rank of BP is reflected by the size of the letters. The lncRNA~mRNA co-expression analysis was used to identify the BPs that were associated with AR-lncRNAs. (I) The BPs that are associate with the AR-lncRNAs in eWAT. Each row indicates a BP while each column represents an AR-lncRNA. Top curve graph represents the distribution of the number of BP associated with the corresponding AR-lncRNA. Right curve graph indicates the distribution of the number of AR-lncRNAs associated with the corresponding BP.

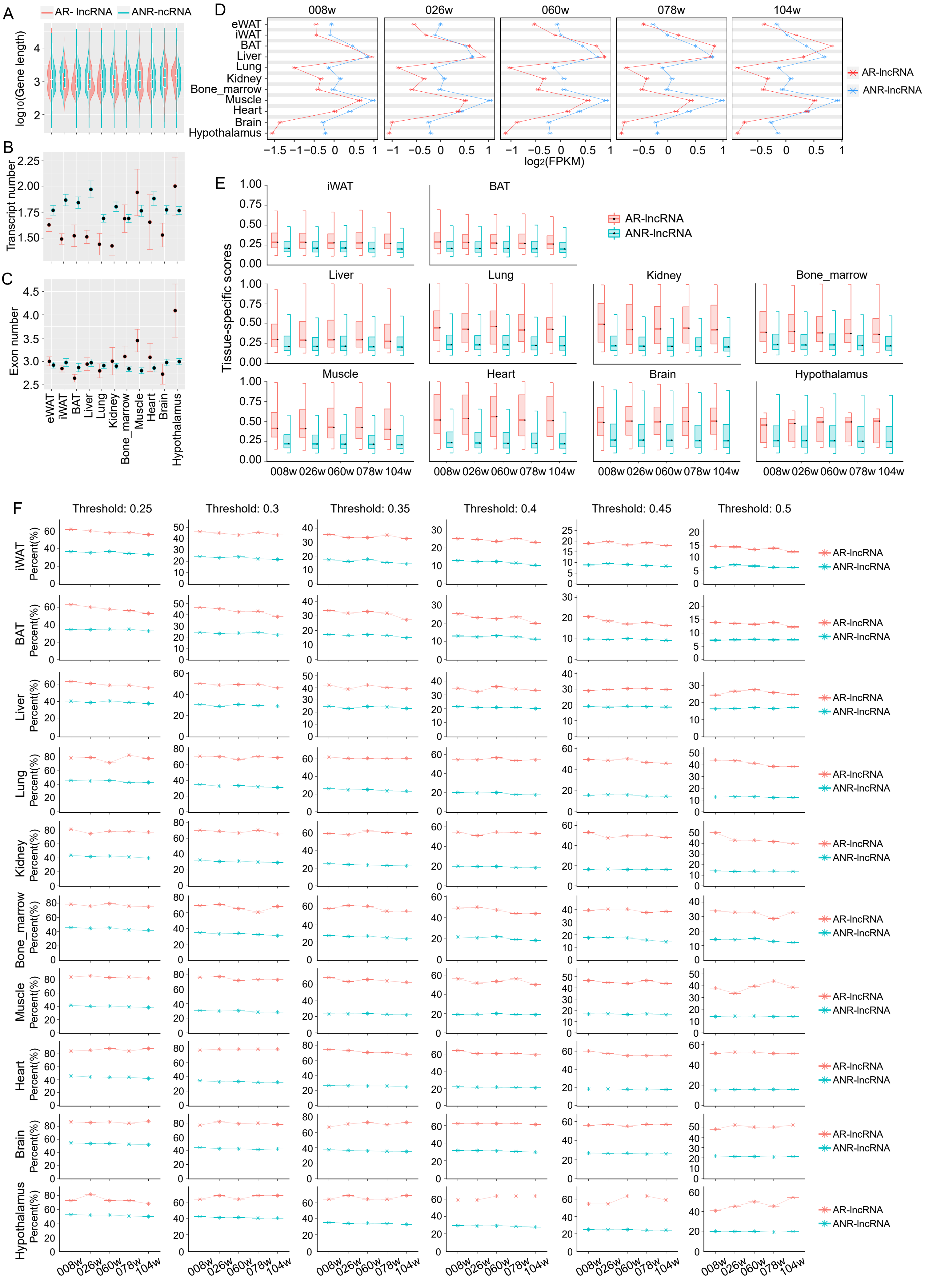

A
AR- lncRNA
ANR-ncRNA
log10(Gene length)
B
Transcript number
C
Exon number
eWAT
iWAT
BAT
Liver
Lung
Kidney
Bone_marrow
Muscle
Heart
Brain
Hypothalamus
D
008w
026w
060w
078w
104w
log2(FPKM)
AR-lncRNA
ANR-lncRNA
E
Tissue-specific scores
iWAT
BAT
Liver
Lung
Kidney
Bone_marrow
Muscle
Heart
Brain
Hypothalamus
AR-lncRNA
ANR-lncRNA
F
Threshold: 0.25
Threshold: 0.3
Threshold: 0.35
Threshold: 0.4
Threshold: 0.45
Threshold: 0.5
Percent(%)
AR-lncRNA
ANR-lncRNA

Figure S3. Gene length, transcript number, exon number, expression abundance, and tissue-specificity between AR-lncRNAs and ANR-lncRNAs across all examined organs, (Related to Figure 3)

(A) Gene length, (B) Transcript number, and (C) Exon number between AR-lncRNAs and ANR-lncRNAs across all examined organs. (D) Expression abundance of AR-lncRNAs and ANR-lncRNAs across the mouse lifespan. The star indicates the median expression of AR-lncRNAs and ANR-lncRNAs. (E) Tissue-specificity of AR-lncRNAs and ANR-lncRNAs in iWAT, BAT, Liver, Lung, Kidney, Muscle, Bone marrow, Heart, Brain, and Hypothalamus. (F) Percentage of tissue-specific lncRNA in AR-lncRNAs and ANR-lncRNAs under varying thresholds of tissue-specific scores. The percentage of tissue-specific lncRNA in AR-lncRNAs and ANR-lncRNAs was calculated under the tissue-specific score thresholds of 0.25, 0.3, 0.35, 0.4, 0.45, and 0.5 across all age stage of 8, 26, 60, 78, and 104 weeks for each organ.

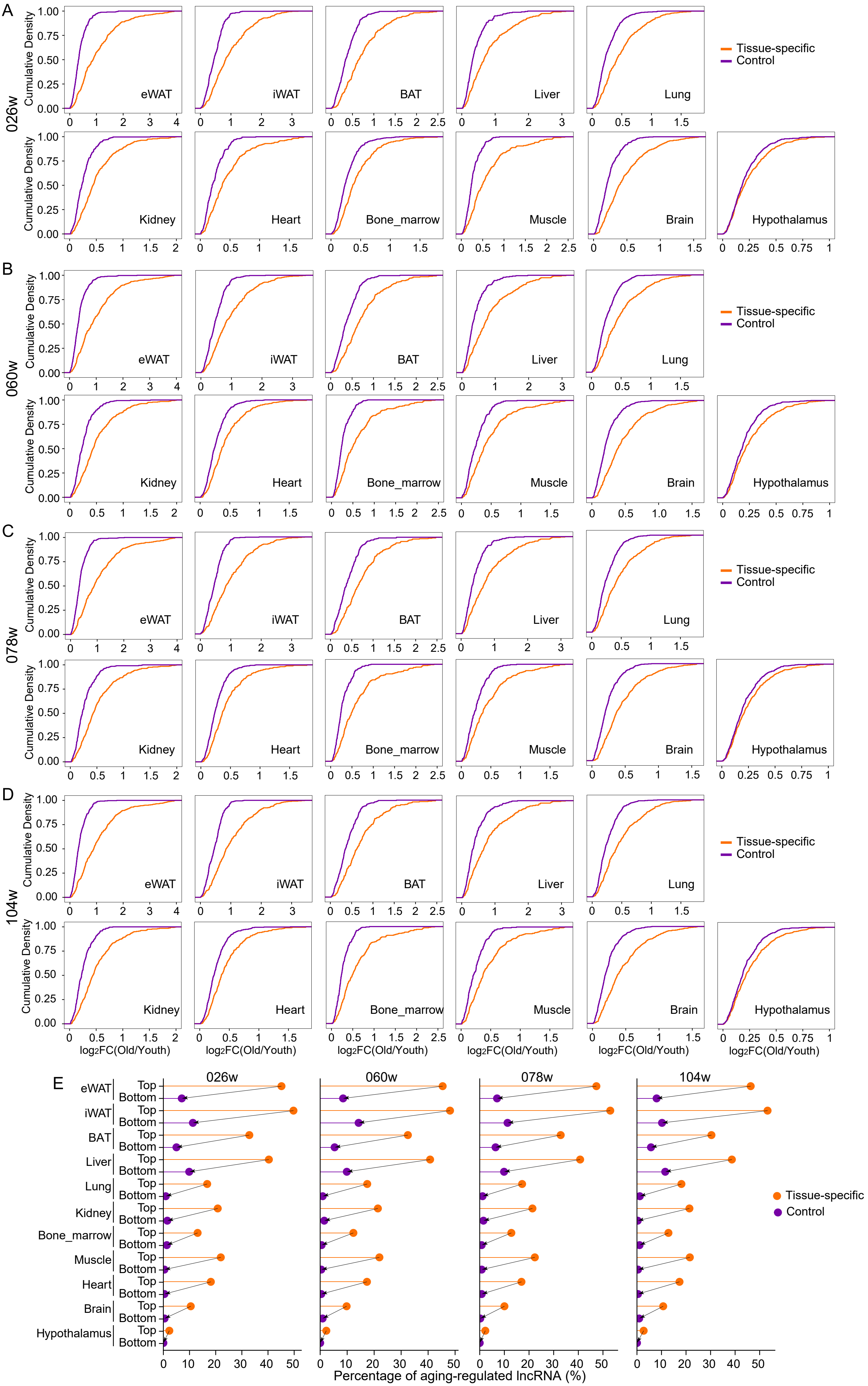
A
B
C
D
E
026w
060w
078w
104w
Cumulative Density
eWAT
iWAT
BAT
Liver
Lung
Kidney
Heart
Bone_marrow
Muscle
Brain
Hypothalamus
Tissue-specific
Control
log2FC(Old/Youth)
Top
Bottom
Percentage of aging-regulated lncRNA (%)

Figure S4. Aging regulation of the tissue-specific and control lncRNAs, (Related to Figure 3)
Log_2FC(Old/Young) is the max log_2(Fold Change, FC) between 78 weeks vs. 8 weeks and 104 weeks vs. 8 weeks. The tissue-specific and control lncRNAs are top 20% and bottom 20% lncRNAs ranked by lncRNAs' tissue-specific scores at the age stage of (A) 26 weeks, (B) 60 weeks, (C) 78 weeks, and (D) 104 weeks. (E). Percentage of AR-lncRNAs between tissue-specific and control lncRNAs across all examined tissues.

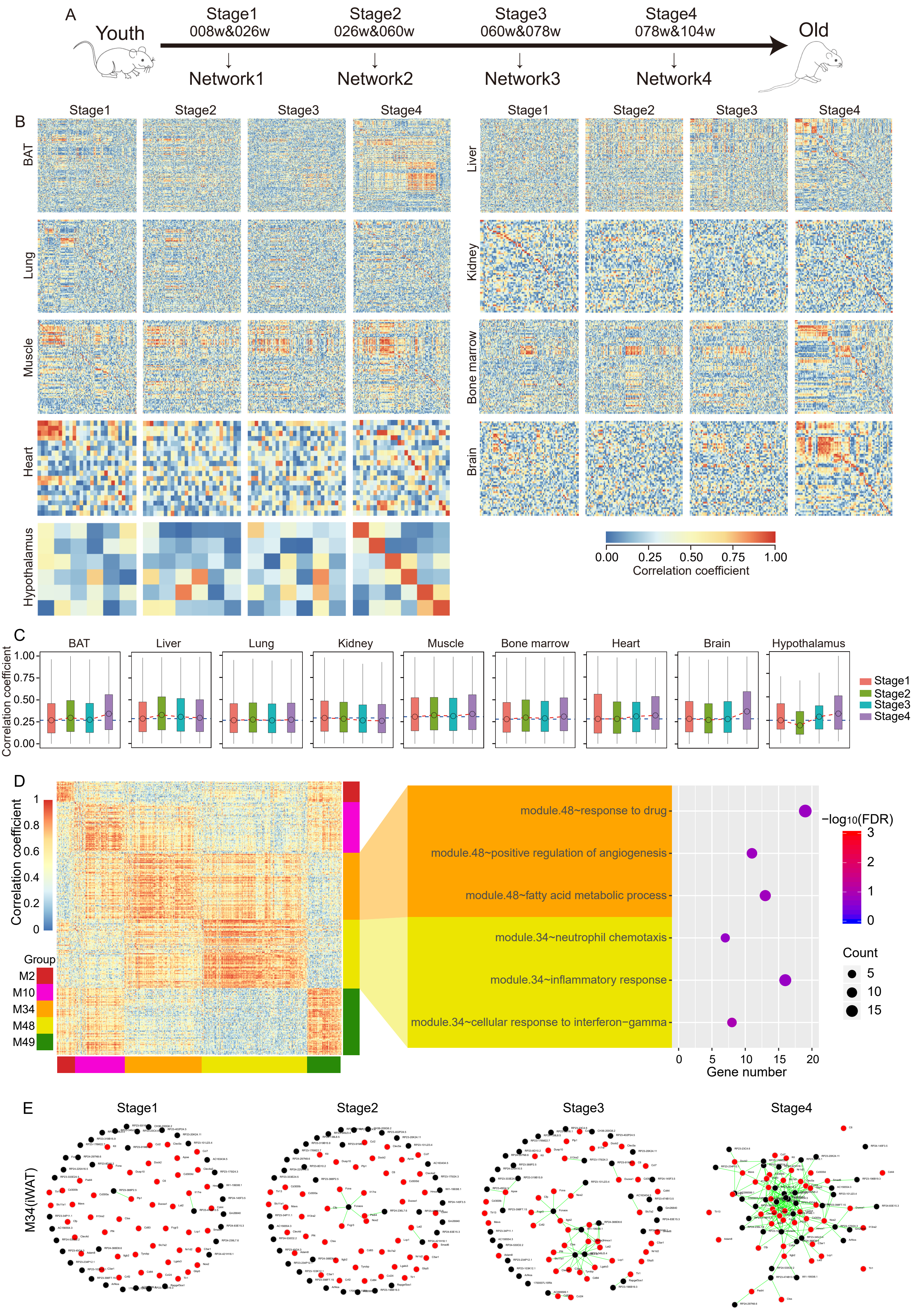

A
Youth
Stage1
008w&026w
Stage2
026w&060w
Stage3
060w&078w
Stage4
078w&104w
Old
Network1
Network2
Network3
Network4
B
Stage1
Stage2
Stage3
Stage4
BAT
Lung
Muscle
Heart
Hypothalamus
Liver
Kidney
Bone marrow
Brain
0.00
0.25
0.50
0.75
1.00
Correlation coefficient
C
BAT
Liver
Lung
Kidney
Muscle
Bone marrow
Heart
Brain
Hypothalamus
Correlation coefficient
Stage1
Stage2
Stage3
Stage4
D
Correlation coefficient
Group
M2
M10
M34
M48
M49
module.48~response to drug
module.48~positive regulation of angiogenesis
module.48~fatty acid metabolic process
module.34~neutrophil chemotaxis
module.34~inflammatory response
module.34~cellular response to interferon−gamma
Gene number
−log10(FDR)
Count
E
Stage1
Stage2
Stage3
Stage4
M34(iWAT)

Figure S5. Aging-regulated lncRNA~mRNA crosstalk across multiple organs, (Related to Figure 4)
(A) Our samples were grouped into 4 aging stages. (B) Heatmap of network between AR-lncRNA and AR-mRNA during aging in BAT, Liver, Lung, Kidney, Muscle, Bone marrow, Heart, Brain, and Hypothalamus. Each row represents an AR-lncRNA while each column represents an AR-mRNA. The color key represents the correlation coefficient between each AR-lncRNA and AR-mRNA comparison. (C) The global correlation coefficients between AR-lncRNAs and AR-mRNAs across mouse lifespan in BAT, Liver, Lung, Kidney, Muscle, Bone marrow, Heart, Brain, and Hypothalamus. The correlation coefficients between AR-lncRNAs and AR-mRNAs were split into quartiles in each stage. The box starts from the first quartile (Q1) to the third quartile (Q3). The second quartile (Q2) was indicated by a circle line in the box. (D) The heatmap of all consensus modules with more than 30 genes in iWAT (left) and functional enrichment of these modules' genes (middle). The number of genes involved in each biological pathway (right). (E) The dynamic changes of AR-lncRNA~AR-mRNA interactions for the genes related to inflammatory pathways in the module (M34) of iWAT.
